# Effect of alloy element on weld pool dynamics in laser welding of aluminum alloys

**DOI:** 10.1038/s41598-018-31350-4

**Published:** 2018-08-28

**Authors:** Masanori Miyagi, Hongze Wang, Ryohei Yoshida, Yousuke Kawahito, Hiroshi Kawakami, Takahisa Shoubu

**Affiliations:** 1 Research & Development Group, Hitachi, Ltd., 7-1-1 Omika, Hitachi, Ibaraki, 319–1292 Japan; 2 Osaka University, JWRI, Osaka, Japan; 3 Mie University, Graduate School of Engineering, 1577 Kurimamachiya-cho, Tsu, Mie 514–8507 Japan; 4 Japan Atomic Energy Agency, Sector of Nuclear Science Research Materials Sciences Research Center, 1-1-1 Kouto, Sayo-cho, Sayo-gun, Hyogo, 679–5148 Japan

## Abstract

In this manuscript, weld pool dynamics in laser welding of various series of aluminum alloys were investigated by the *in situ* X-ray phase contrast imaging system. The experimental results showed that metal irradiated by laser was evaporated immediately, which generated the keyhole. Then metal surrounding the keyhole was melted gradually with the heat from keyhole. The growth rate of keyhole depth had a positive linear correlation with the total content of low boiling temperature elements (TCE), so did the keyhole depth and diameter at the stable stage. Longitudinal view area of the molten pool had a negative linear correlation with the thermal conductivity of aluminum alloy. The measured laser absorption rate had the same variation trend with the ratio of keyhole depth to diameter, and the highest absorption rate of 58% appeared in laser welding of aluminum alloy with TCE equal to 2.1%. Violent fluctuation in keyhole shape was avoided in aluminum alloy with TCE lower than 2.1%, where the surface tension and recoil pressure of metal vapor were balanced. To sum up, the effect of alloy element on weld pool dynamics in laser welding of aluminum alloys was firstly quantified in this manuscript.

## Introduction

Aluminum alloy is one of key materials in weight saving of mobile body including electric vehicle (EV). Laser manufacturing has been widely used as a highly efficient method to achieve high quality product in industry^1,2^, where laser is used in welding^3–5^, cutting^6,7^, additive manufacturing^8^, and so on. The interaction between laser and material determines the product quality^9–13^. However, keyhole and molten metal induced by high power density laser are surrounded by solid metal, and these inner phenomena are not observable with the conventional method. Though characteristics of keyhole and molten pool were presented by simulation^14–17^, these calculated results have rarely been validated by experiment directly.

Various methods have been adopted to explore the dynamic behavior inside weld pool in laser material processing^18–21^. High speed camera was adopted to observe the movement of molten metal in the weld pool with a specially designed structure^18,19,22^. In this method, a glass sheet and a steel sheet were arranged side by side, and their interface was irradiated by laser. By placing a camera at the glass side, it is possible to directly capture the inside behavior of the weld pool, which contributed a better understanding of the keyhole dynamics and flow of the molten pool. However, this specially designed structure changed the heat conduction condition around the weld pool, and the measured keyhole size was clearly larger than the real one in laser processing. Microfocused X-ray transmission *in situ* observation system was successfully adopted to investigate the keyhole dynamics^20,21^, which contributed an in-depth understanding of the light absorption^23^, stability of the keyhole^24^ in laser welding. However, the interface between molten pool and solid metal couldn’t been identified with this method due to the marginal difference in absorption performance of X-ray between liquid phase and solid phase. Recently, the X-ray phase contrast imaging system was reported to have the ability to capture the weld pool behavior and porosity formation process clearly^25,26^, which firstly provided an effective method to quantify the real, inner characteristics in welding.

Researchers have focused on the weld quality in laser welding of various series of aluminum alloys by both experiments and simulations^27–29^. These previous researches showed that the aluminum alloy with different compositions had different weldability, which was caused by the differences in weld pool behavior. The latest paper published by John H. martin *et al*. in Nature showed that the composition of aluminum alloy including the nanoparticles of nucleants would significantly affect the quality of final product in laser manufacturing^30^. Several researchers found that Mg element in aluminum alloy evaporated during the laser welding process, which would affect the characteristics of keyhole dynamics, and the final properties of the weld bead, e.g. hardness distribution, welding defect including undercut and porosity^31–35^. However, the relationship between aluminum alloy with different compositions and behavior of weld pool induced by laser is still not fully understood.

In this manuscript, X-ray phase contrast imaging system was adopted to capture the weld pool behavior and porosity formation process in laser welding of various series of aluminum alloys. Effect of alloy composition on keyhole growth rate, keyhole geometry and absorption rate was discussed. The relationship between longitudinal view area of the weld pool and thermal conductivity of the material was presented. This paper provided a quantitative research about effect of alloy element on weldability of aluminum.

## Results and Discussion

**Figure 1:**
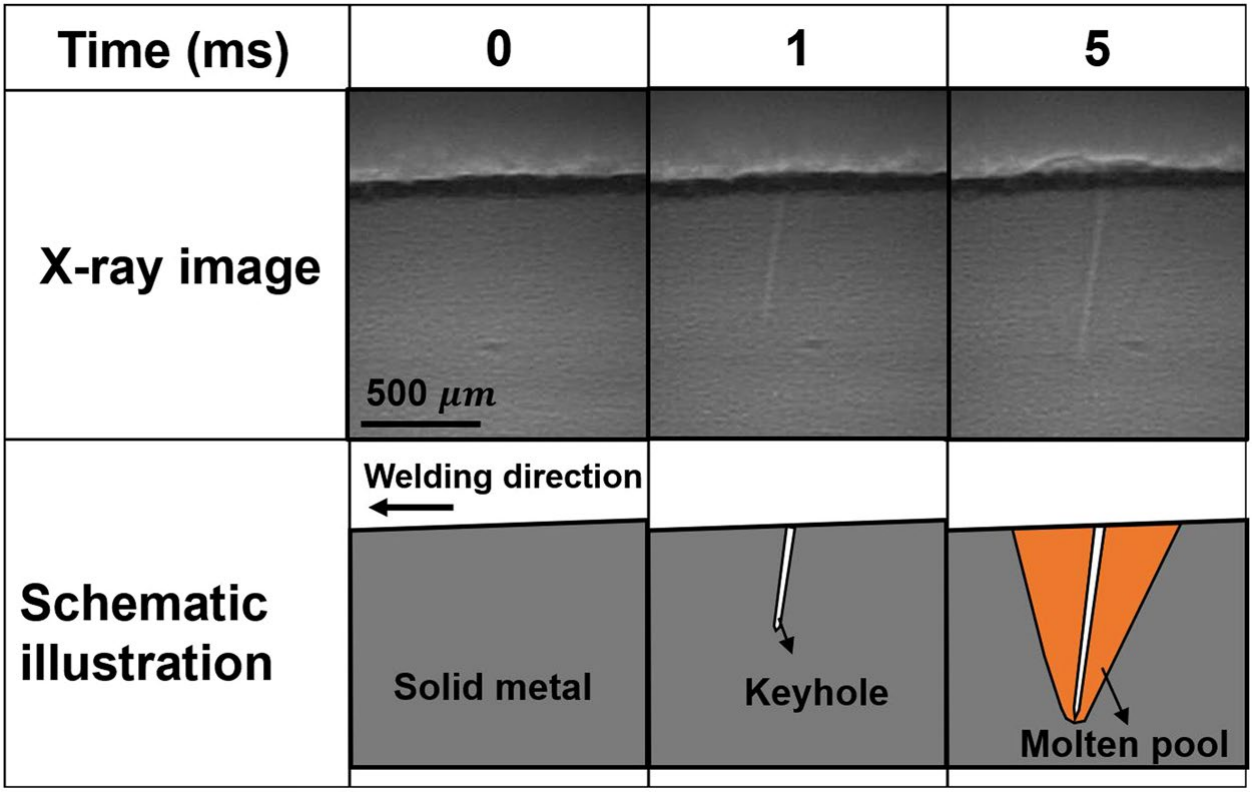
Dynamic longitudinal view of weld pool during pool formation process captured by X-ray imaging system (A1050).

Keyhole and molten pool were captured by X-ray phase contrast imaging system to reveal the quantitative relationship between the observed characteristics and composition of aluminum alloys. Figure 1 shows the captured longitudinal behavior of A1050 weld pool during pool formation process. At 1 ms, keyhole with large ratio of depth to diameter was generated, while molten pool surrounding the keyhole was not obvious, which indicated that keyhole growth rate was higher than the molten pool growth rate at this time. At the time of 5 ms, molten pool with large area surrounding the keyhole appeared. During this process, heat was transferred from metal at the wall of keyhole to metal surrounding the keyhole, and molten metal surrounding the keyhole was gradually formed. The captured behavior of weld pool proved that heat of laser was firstly absorbed in the keyhole, then transferred to metal surrounding the keyhole.

**Figure 2:**
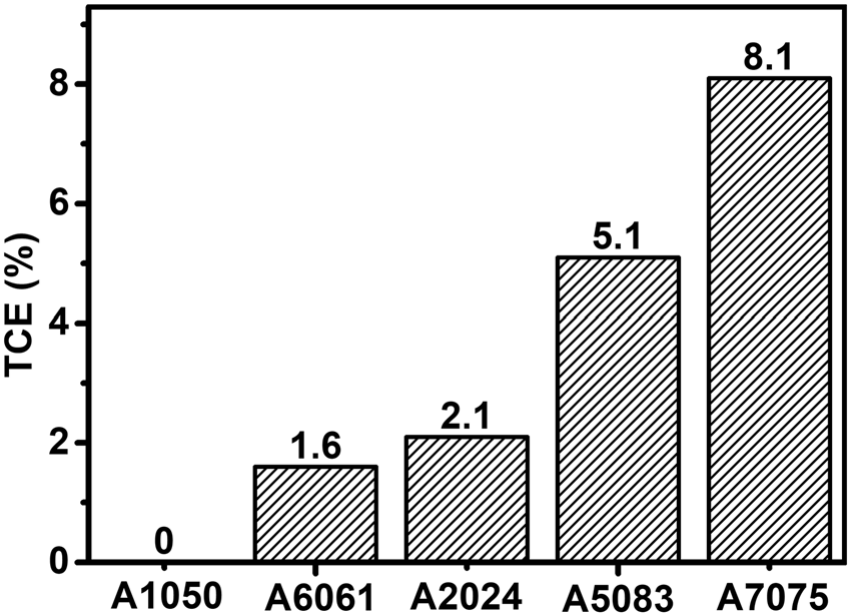
Total content of low boiling temperature elements (TCE) varying with the grade of aluminum alloys.

Composition of aluminum alloys can have a significant effect on thermal properties, e.g. boiling temperature, thermal conductivity. Thus, behavior of weld pool may vary with the composition of aluminum alloys. It is important to quantify the effect of composition. The content of element with boiling temperature lower than that of aluminum element can significantly affect the dynamic behavior of keyhole^27^. The total content of low boiling temperature elements (TCE), which was defined to be the total content of the elements with the boiling temperature lower than that of aluminum (Si, Mn, Mg, and Zn in present work), was calculated, and the result is shown in Fig. 2. As shown, TCE varies with the grade of aluminum. A1050 has the lowest TCE with the value of 0.0%, while A7075 has the highest TCE with the value of 8.5%.

**Figure 3:**
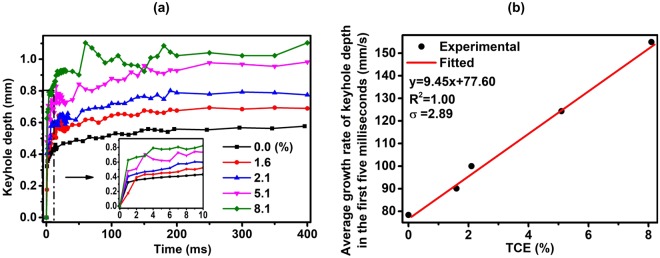
Dynamic keyhole depth during the molten pool formation process: (**a**) time-dependent variation in keyhole depth for representative aluminum alloys; (**b**) relationship between the average growth rate of keyhole depth at the first 5 ms and TCE.

During the keyhole and molten pool formation process, keyhole depth increased with time. The dynamic process of keyhole depth when laser irradiating to several representative aluminum alloys was captured by X-ray phase contrast imaging system, and the result is shown in Fig. 3(a). To highlight the relationship between TCE and keyhole depth, the corresponding TCE value of aluminum alloy for each keyhole depth variation curve was labeled in the figure. As shown, the keyhole depth increased rapidly in the first 5 ms, then the increasing rate decreased slowly to 0, thus, the keyhole depth reached a stable value with small fluctuation. The keyhole depth at the stable stage for different aluminum alloys increased with TCE.

The average growth rate of keyhole depth at the first 5 ms for each aluminum alloy was calculated, and the relationship between the average growth rate of keyhole depth and TCE is shown in Fig. 3(b). As shown, the average growth rate increased with TCE. Linear fitting was conducted to quantify the relationship between the average growth rate and TCE, and the fitting results are shown together with the experimental results. Assuming that y represents the average growth rate of keyhole depth, and x represents TCE, the fitted relationship between y and x is y = 9.45x + 77.60, σ = 2.89 and R^2^ value is 1.00. The fitted R^2^ value indicates that a perfect positive linear correlation exists between the average growth rate and TCE. The element with the boiling temperature lower than that of Al will evaporate earlier before Al when heated by laser, which will accelerate the formation and increase the depth of keyhole. This can explain why the growth rate of keyhole depth will increase with TCE. Zhou *et al*. (2017) conducted spectroscopic analysis to quantify the composition of metal vapor in laser welding of A5052, and the results indicated that a large amount of Mg was evaporated during the welding process^29^. This research supports our finding that the element with boiling temperature lower than that of aluminum plays a significant role on keyhole formation. Thus, TCE can be utilized as a pointer for the speed to form the keyhole.

**Figure 4:**
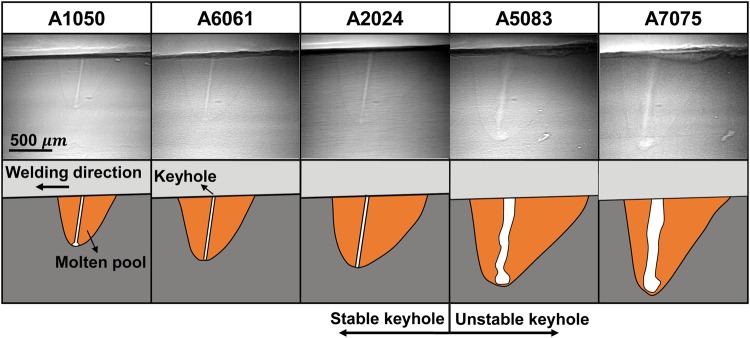
Representative longitudinal view of molten aluminum alloys pool at the stable stage.

Figure 4 shows the representative longitudinal characteristics of keyhole and molten pool for representative aluminum alloys at the stable stage. There was almost no molten metal at the bottom of the keyhole, and the keyhole depth was equal to the molten pool depth. The weld pool area in front of the keyhole was smaller than that behind the keyhole. In the weld pools of A1050, A6061 and A2024 (TCE is 0.0%, 1.6%, and 2.1%, respectively), keyhole diameter varied little from top to bottom, and keyhole was stable. In the molten pool of A5083 and A7075 (TCE was 5.1% and 8.1%, respectively), keyhole shape varied violently. These experimental results indicated that violent fluctuation of the keyhole can be prevented when TCE was lower than 2.1%, where the surface tension and recoil pressure of metal vapor were balanced.

**Figure 5:**
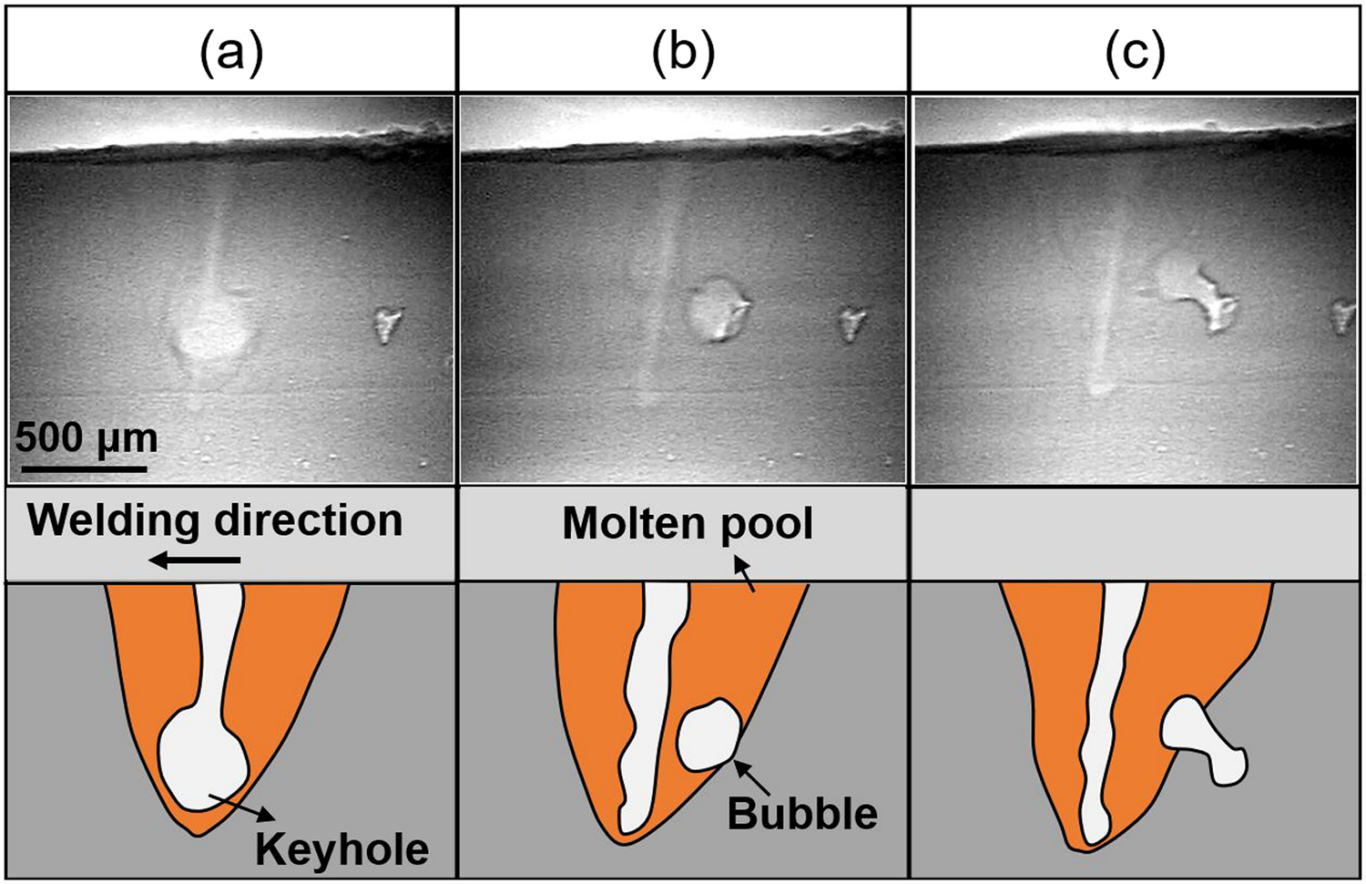
Representative longitudinal view of the A7075 molten pool.

Figure 5 shows the supplementary longitudinal view of the molten pool in laser welding of A7075, which provides a detailed description about the violent fluctuation in keyhole shape. Three representative characteristics during the welding process are presented. Though the keyhole depth and diameter at the inlet didn’t have an obvious variation, shape of the keyhole varied with time during the welding process. At a time, diameter of the keyhole varied with the depth. In Fig. 5(a), keyhole diameter at the bottom position is approximately three times of that at the inlet. In Fig. 5(b), a large bubble was formed in the molten metal. In Fig. 5(c), a large bubble appeared at the boundary between weld pool and solidified zone. The bubbles induced by the dynamic keyhole would lead to the formation of porosity in the weld seam.

**Figure 6:**
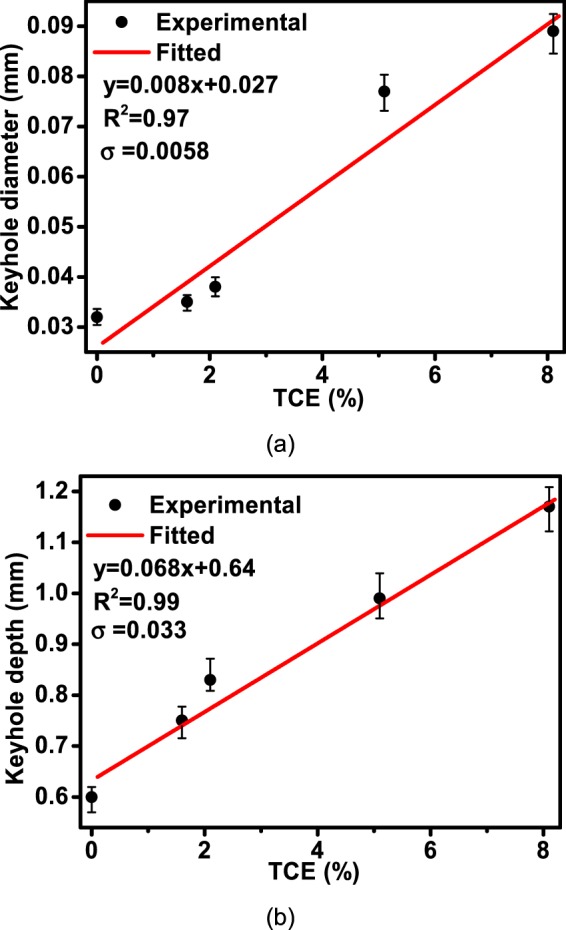
Relationship between TCE and: (**a**) keyhole diameter; (**b**) keyhole depth.

The relationship between keyhole diameter at the stable stage and TCE is shown in Fig. 6(a). For the keyhole with diameter varying with depth dynamically in laser welding of A5083 and A7075, the average diameter of keyhole at the inlet was adopted. We measured five inlet diameters of the keyhole at five random time of the stable stage, and the average value was calculated. As shown, keyhole diameter increases with TCE. Linear fitting was conducted to quantify the relationship between the keyhole diameter and TCE, and the fitting results are shown together with the experimental results. Assuming that y represents the keyhole diameter, and x represents TCE, the fitted relationship between y and x is y = 0.008x + 0.027, σ = 0.0058 and R^2^ value is 0.97. The fitted R^2^ value indicates that good linear relationship exists between the keyhole diameter and TCE.

The relationship between keyhole depth at the stable stage and TCE is shown in Fig. 6(b). As shown, keyhole depth increases with TCE. Linear fitting was conducted to quantify the relationship between the keyhole depth and TCE, and the fitting results are shown together with the experimental results. Assuming that y represents the keyhole depth, and x represents TCE, the fitted relationship between y and x is y = 0.068x + 0.64, σ = 0.033 and R^2^ value is 0.99. The fitted R^2^ value indicates that good linear relationship exists between the keyhole depth and TCE. With the increase of TCE in aluminum alloy, the amount of low boiling temperature element in the metal surrounding the keyhole increased, and this part of element would evaporate during the welding process randomly, which increased both the diameter and the instability of the keyhole. This randomly produced metal vapor made of low boiling temperature element changed the shape of the keyhole, and keyhole diameter varied violently.

**Figure 7:**
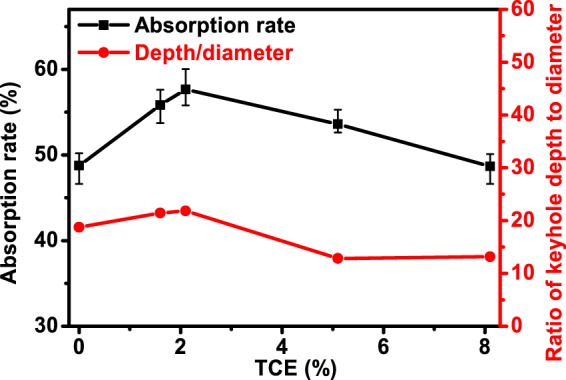
Effect of TCE on absorption rate and ratio of keyhole depth to diameter.

The absorption rate in laser welding of representative aluminum alloys was measured, and the result is shown in Fig. 7. The absorption rate increased with TCE at first when TCE was lower than 2.1%, then decreased with TCE. The highest absorption rate of 57.7% appeared when TCE was 2.1%. To quantify the key factor that affecting the absorption rate, the ratio of keyhole depth to diameter for each aluminum alloy was calculated based on the experimental results shown in Fig. 6, and the result is shown together with the measured absorption rate. As shown, the absorption rate had the same variation trend with the ratio of keyhole depth to diameter. Previous research reported that the ratio of keyhole depth to diameter was an indicator for the times of multiple reflections in the keyhole^21^. With the increase of the ratio, the times of multiple reflections in the keyhole would increase, which contributed to the increase in absorption rate. The absorption rate at large TCE value was abnormal. Though the ratios of keyhole depth to diameter at the TCE of 5.1% and 8.5% were lower than that at the TCE of 0%, the absorption rates were higher. That was because the fluctuation in keyhole shape increased the times of multiple reflections, thus increased the absorption rate.

**Figure 8:**
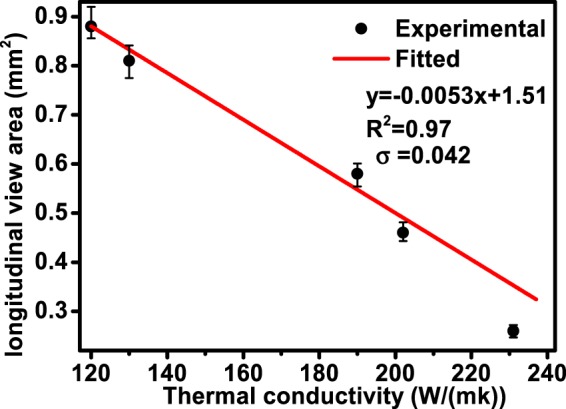
Relationship between longitudinal view area of the molten metal and thermal conductivity.

The relationship between longitudinal view area of the molten pool at the stable stage and thermal conductivity of the aluminum alloy is shown in Fig. 8. As shown, longitudinal view area of the molten pool decreased with thermal conductivity. Linear fitting was conducted to quantify the relationship between the section area and thermal conductivity, and the fitting result is shown together with the experimental results. Assuming that y represents the section area, and x represents thermal conductivity, the fitted relationship between y and x is y = −0.0053x + 1.51, σ = 0.042 and R^2^ value is 0.97. The fitted R^2^ value indicates that good linear relationship exists between the longitudinal view area and the thermal conductivity. Longitudinal view area of the molten pool is generally determined by both energy absorption and energy dissipation process, this interaction process is so complicated that there is no easy method to quantify this process at present. The experimental linear relationship between the longitudinal view area and the thermal conductivity contributes to understanding of key factor that determining the size of the molten pool.

**Figure 9:**
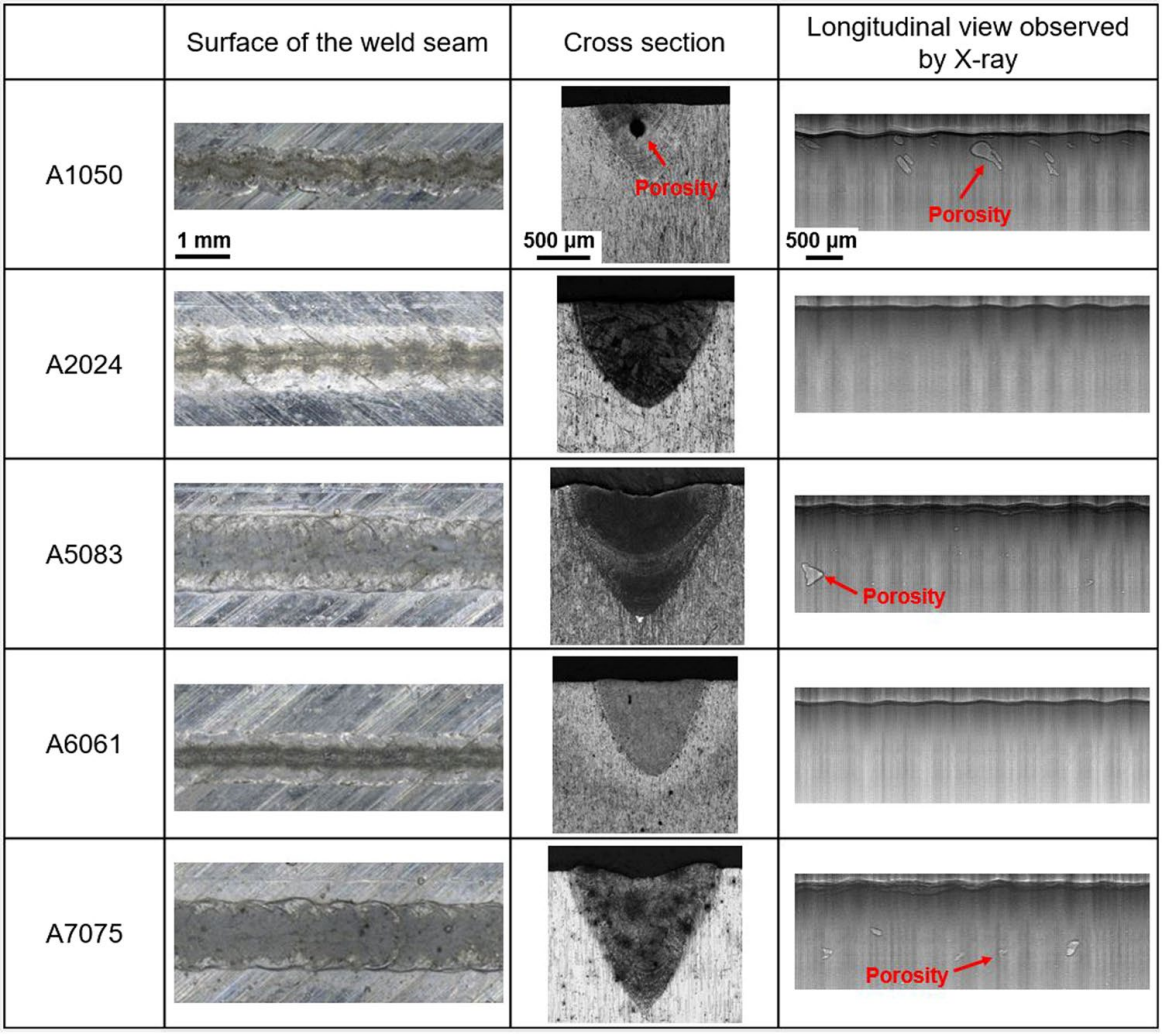
Characteristics of surface, cross section and longitudinal view in laser-welded aluminum alloys weld seam.

Figure 9 shows the characteristics of surface, cross section and longitudinal view in laser-welded aluminum alloys weld seam. Characteristics of the surface and cross section were measured by optical microscope. The results validated that width, depth and cross section area of the weld seam varied with the aluminum alloy, and this variation law was consistent with that captured by X-ray imaging system shown in Fig. 4. Longitudinal view of the weld seam was also captured by X-ray phase contrast imaging, as shown in Fig. 9. There was rarely any porosity in the weld seam of A6061 and A2024, while large porosities were observed in the weld seam of A1050, A5083 and A7075. These results about the porosities were also consistent with the *in situ* observed bubbles distribution in the weld pool. Bubbles in the molten metal can be affected by a lot of factors, e.g. composition of the alloy, laser parameters including power, speed and beam diameter, shielding gas, and so on^13,25,36^. The mechanism for bubble formation in the weld pool and the method to suppress the bubble will be investigated in the future.

## Conclusion

1. Longitudinal view of the weld pool in laser welding was observed by the X-ray phase contrast imaging system. Keyhole was immediately generated when laser irradiated to aluminum alloy, then material surrounding the keyhole was melted gradually with the heat from keyhole. The keyhole depth was equal to the molten pool depth.
2. The growth rate of keyhole depth had a positive linear correlation with the total content of low boiling temperature elements (TCE), so did the keyhole depth and diameter at the stable stage. Violent fluctuation in keyhole shape was avoided in aluminum alloy with TCE lower than 2.1%.
3. The measured laser absorption rate had the same variation trend with the ratio of keyhole depth to diameter, and the highest absorption rate of 57.7% appeared when TCE was 2.1%.
4. Longitudinal view area of the molten pool had a negative linear correlation with the thermal conductivity of aluminum alloy.

## Methods

With X-ray phase contrast imaging system, an image with emphasized edge of weld pool is captured based on the observation of interference patterns between diffracted and undiffracted waves^25,26^. Because this imaging system puts forwards a high requirement for the X-ray beam to generate the clear interference patterns, the high quality BL22XU beam line at SPring-8 was utilized.

**Figure 10:**
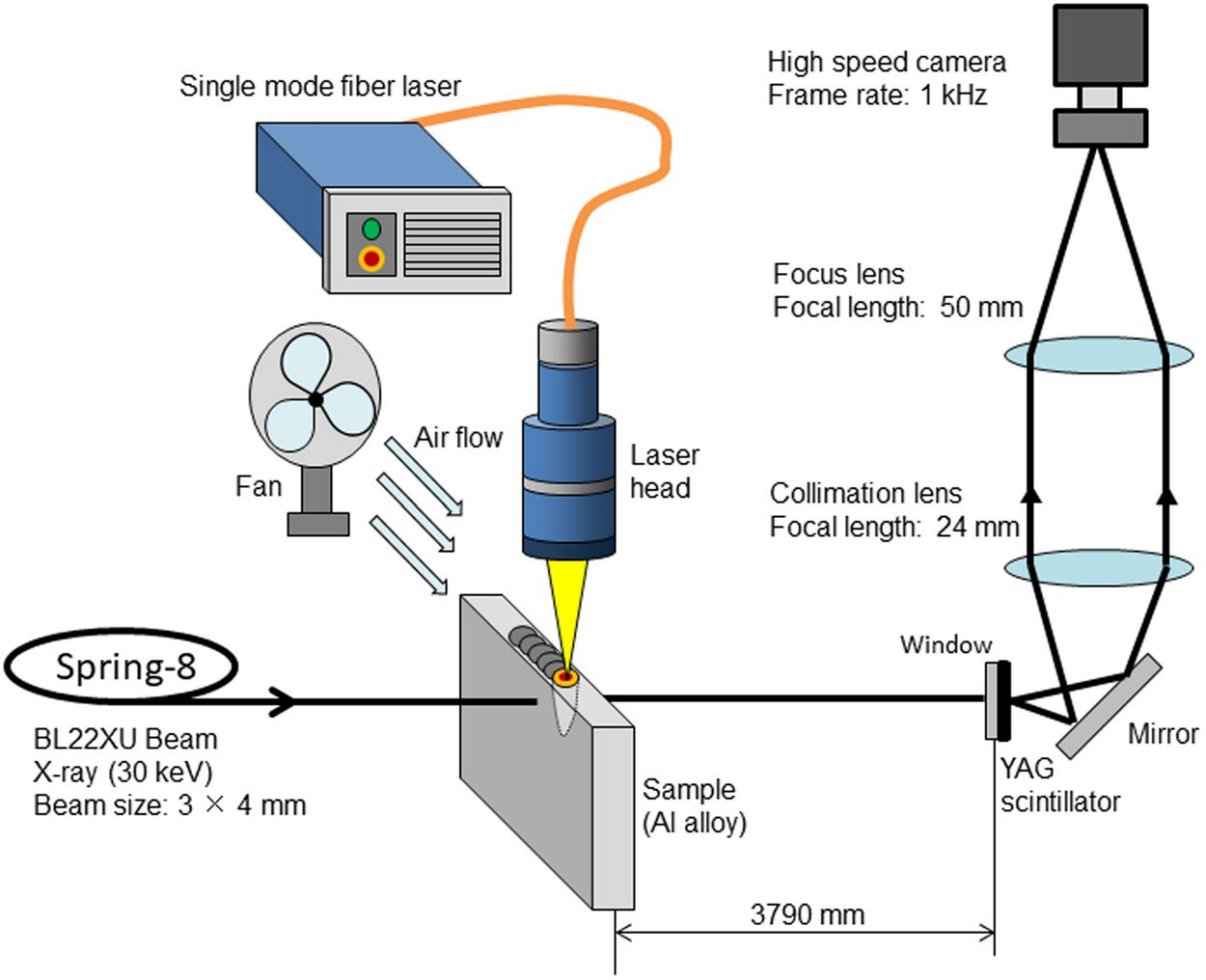
Imaging system developed in our lab.

**Figure 11:**
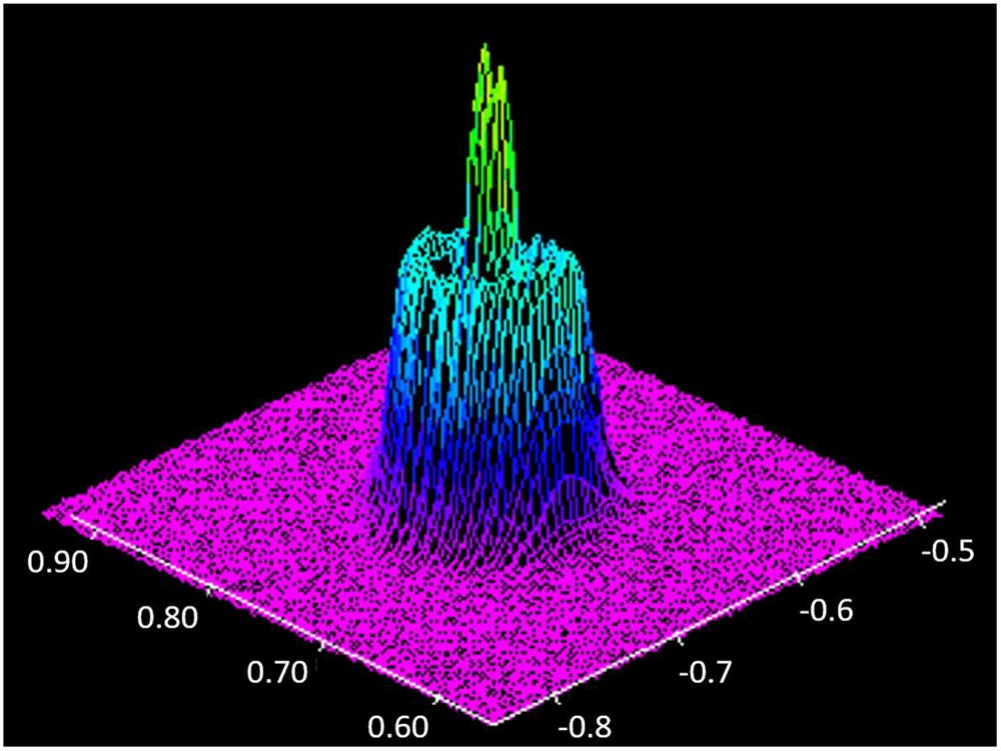
Intensity profile of laser beam (defocus distance: −1 mm).

**Figure 12:**
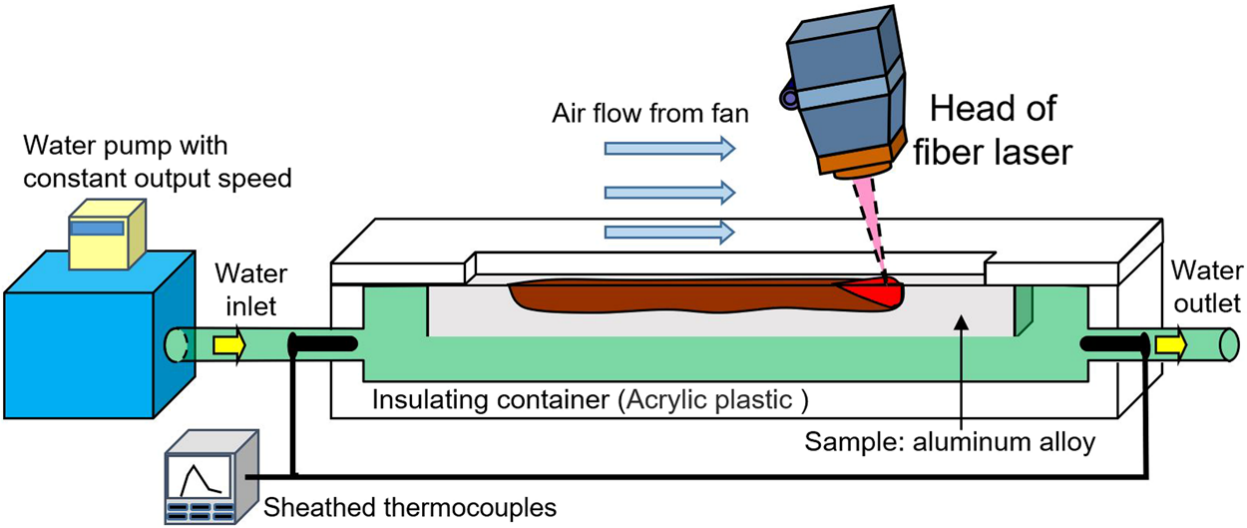
Schematic of the equipment to measure the absorption rate.

The *in situ* imaging system is shown in Fig. 10. The geometry of the samples was 70 × 30 × 3 (mm). X-ray generated in BL22XU beam line was irradiated to side surface of the aluminum alloy sheet, laser beam was applied to the upper surface (70 × 3 (mm) surface). The capturing frequency of the high speed camera was 1.0 kHz. Five representative aluminum alloys were used in the experiment, which were A1050, A2024, A5083, A6061, and A7075, respectively. Element composition of each alloy is listed in Table 1^37^. The power of single-mode fiber laser^26^ in the experiment was 500 W, welding speed was 16.7 mm/s, and defocus distance was −1 mm. The 10° inclination angle of laser beam was adopted to reduce the reflection. Figure 11 shows the intensity profile of laser beam at the defocus of −1 mm. The diameter of the laser beam at this welding condition was 140 μm. This specific laser-beam condition was selected because the maximum observation area for the SPring-8 X-ray source was 1 × 1 mm. A fan was used to remove the metallic plume during the experiment^25^. Table 2 shows thermal conductivity of the aluminum alloys at room temperature^38^. Because there are significant composition differences between these aluminum alloys, thermal conductivity varies with the grade. Table 3 shows the boiling temperature of the main elements in aluminum alloy^39^. The boiling temperatures for Si, Mn, Mg and Zn element were lower than that of aluminum. To quantify the relationship between keyhole shape and energy absorption, the laser-light absorption rate of each aluminum alloy during the welding process was measured with the water calorimetric method^21,28^. The experimental system to measure the absorption rate is shown in Fig. 12. During the welding process, the aluminum heated by laser was cooled by the flowing water, and the heat transferred to the water was assumed to be approximately equal to the heat absorbed by aluminum. After the increase of heat in the water was calculated based on the measured temperature gap and mass, the absorption rate of laser could be obtained.

**Table 1:** Element composition of aluminum alloys (mass %).

	Al	Si	Cu	Mn	Mg	Cr	Zn
A1050	99.5 (min)	—	—	—	—	—	—
A2024	93.5	—	4.4	0.6	1.5	—	—
A5083	94.7	—	0.05	0.7	4.4	0.15	—
A6061	97.9	0.6	0.28	—	1.0	0.20	—
A7075	89.0	—	1.6	—	2.5	0.23	5.6

**Table 2:** Thermal conductivity of aluminum alloys at room temperature.

	A1050	A2024	A5083	A6061	A7075
Thermal conductivity (W/(mK))	231	190	120	202	130

**Table 3:** Boiling temperature of elements in aluminum alloy.

	Al	Si	Cu	Mn	Mg	Cr	Zn	Zr
Boiling temperature (K)	2743	2633	2868	2373	1383	2755	1180	3853

## References

[1] David S, DebRoy T (1992). Current issues and problems in welding science. Science.

[2] Hofmann, D. C. *et al*. Developing gradient metal alloys through radial deposition additive manufacturing. *Scientific reports* **4** (2014). 10.1038/srep05357 PMC4062900 24942329

[3] Ma C, Chen L, Cao C, Li X (2017). Nanoparticle-induced unusual melting and solidification behaviours of metals. Nature communications.

[4] Wang L, Gao M, Zhang C, Zeng X (2016). Effect of beam oscillating pattern on weld characterization of laser welding of AA6061-T6 aluminum alloy. Materials & Design.

[5] Wang H, Zhang Y, Li S (2016). Laser welding of laminated electrical steels. Journal of Materials Processing Technology.

[6] Witte U (2016). kW-class direct diode laser for sheet metal cutting based on DWDM of pump modules by use of ultra-steep dielectric filters. Optics Express.

[7] Chen C, Gao M, Zeng X (2016). Relationship between temperature at cut front edge and kerf quality in fiber laser cutting of Al–Cu aluminum alloy. International Journal of Machine Tools and Manufacture.

[8] DebRoy, T. *et al*. Additive manufacturing of metallic components–Process, structure and properties. *Progress in Materials Science* (2017).

[9] Kaplan A (2012). Absorptivity modulation on wavy molten steel surfaces: The influence of laser wavelength and angle of incidence. Applied Physics Letters.

[10] Fabbro R (2010). Melt pool and keyhole behaviour analysis for deep penetration laser welding. Journal of Physics D: Applied Physics.

[11] Zhang L, Zhang J, Gumenyuk A, Rethmeier M, Na S (2014). Numerical simulation of full penetration laser welding of thick steel plate with high power high brightness laser. Journal of materials processing technology.

[12] Pang S, Chen W, Wang W (2014). A quantitative model of keyhole instability induced porosity in laser welding of titanium alloy. Metallurgical and Materials Transactions A.

[13] Lin R, Wang H-p, Lu F, Solomon J, Carlson BE (2017). Numerical study of keyhole dynamics and keyhole-induced porosity formation in remote laser welding of Al alloys. International Journal of Heat and Mass Transfer.

[14] Wei H, Elmer J, DebRoy T (2017). Crystal growth during keyhole mode laser welding. Acta Materialia.

[15] Wei H, Elmer J, DebRoy T (2017). Three-dimensional modeling of grain structure evolution during welding of an aluminum alloy. Acta Materialia.

[16] Pang S, Chen L, Zhou J, Yin Y, Chen T (2010). A three-dimensional sharp interface model for self-consistent keyhole and weld pool dynamics in deep penetration laser welding. Journal of Physics D: Applied Physics.

[17] Zhao H (2011). Modelling of keyhole dynamics and porosity formation considering the adaptive keyhole shape and three-phase coupling during deep-penetration laser welding. Journal of Physics D: Applied Physics.

[18] Zhang M, Chen G, Zhou Y, Li S (2013). Direct observation of keyhole characteristics in deep penetration laser welding with a 10 kW fiber laser. Optics express.

[19] Zou J, Ha N, Xiao R, Wu Q, Zhang Q (2017). Interaction between the laser beam and keyhole wall during high power fiber laser keyhole welding. Optics Express.

[20] Matsumoto N, Kawahito Y, Nishimoto K, Katayama S (2017). Effects of laser focusing properties on weldability in high-power fiber laser welding of thick high-strength steel plate. Journal of Laser Applications.

[21] Wang, H., Nakanishi, M. & Kawahito, Y. Effects of welding speed on absorption rate in partial and full penetration welding of stainless steel with high brightness and high power laser. *Journal of Materials Processing Technology* (2017).

[22] Wang H, Nakanishi M, Kawahito Y (2018). Dynamic balance of heat and mass in high power density laser welding. Optics express.

[23] Kawahito Y, Matsumoto N, Abe Y, Katayama S (2011). Relationship of laser absorption to keyhole behavior in high power fiber laser welding of stainless steel and aluminum alloy. Journal of Materials Processing Technology.

[24] Kawahito Y, Mizutani M, Katayama S (2007). Elucidation of high-power fibre laser welding phenomena of stainless steel and effect of factors on weld geometry. Journal of Physics D: Applied Physics.

[25] Miyagi M, Kawahito Y, Kawakami H, Shoubu T (2017). Dynamics of solid-liquid interface and porosity formation determined through x-ray phase-contrast in laser welding of pure Al. Journal of Materials Processing Technology.

[26] Kawahito Y, Wang H (2018). *In-situ* observation of gap filling in laser butt welding. Scripta Materialia.

[27] Katayama S, Nagayama H, Mizutani M, Kawahito Y (2009). Fibre laser welding of aluminium alloy. Weld. Int..

[28] Kawahito Y, Matsumoto N, Abe Y, Katayama S (2012). Laser absorption of aluminium alloy in high brightness and high power fibre laser welding. Weld. Int..

[29] Zhou L, Zhang M, Jin X, Zhang H, Mao C (2017). Study on the burning loss of magnesium in fiber laser welding of an Al-Mg alloy by optical emission spectroscopy. The International Journal of Advanced Manufacturing Technology.

[30] Martin JH (2017). 3D printing of high-strength aluminium alloys. Nature.

[31] Moon D, Metzbower E (1983). Laser beam welding of aluminum alloy 5456. Welding Journal.

[32] Cieslak M, Fuerschbach P (1988). On the weldability, composition, and hardness of pulsed and continuous Nd: YAG laser welds in aluminum alloys 6061, 5456, and 5086. Metallurgical Transactions B.

[33] Xijing, W., Katayama, S. & Matsunawa, A. Character of melting and evaporation in laser beam welding of two aluminum alloys. *Welding journal* **76** (1997).

[34] Pastor M, Zhao H, Martukanitz R, DebRoy T (1999). Porosity, underfill and magnesium lose during continuous wave Nd: YAG laser welding of thin plates of aluminum alloys 5182 and 5754. Welding Journal-New York-.

[35] Dilthey U, Goumeniouk A, Lopota V, Turichin G, Valdaitseva E (2001). Development of a theory for alloying element losses during laser beam welding. journal of physics d: applied physics.

[36] Huang L, Hua X, Wu D, Li F (2018). Numerical study of keyhole instability and porosity formation mechanism in laser welding of aluminum alloy and steel. Journal of Materials Processing Technology.

[37] Davis, J. R. Aluminum and Aluminum Alloys (2013).

[38] Handbook AM (1990). Properties of wrought aluminum and aluminum alloys. ASM International Hand Book Committee.

[39] Zhang Y, Evans JR, Yang S (2011). Corrected values for boiling points and enthalpies of vaporization of elements in handbooks. Journal of Chemical & Engineering Data.

